# Generation of a cleaned dataset listing Avon Longitudinal Study of Parents And Children peer-reviewed publications to 2015

**DOI:** 10.12688/wellcomeopenres.14986.1

**Published:** 2018-12-19

**Authors:** Oliver Butters, Amran Ismail, Sue Thompson, Rebecca Wilson

**Affiliations:** 1 Institute of Health and Society, Newcastle University, UK, Newcastle upon Tyne, UK; 2 Social and Community Medicine, University of Bristol, Bristol, UK

**Keywords:** Birth cohort, Bibliography, ALSPAC

## Abstract

Birth cohort studies generate huge amounts of data, and as a consequence are a source of many peer reviewed publications. We have taken the list of publications from the Avon Longitudinal Study of Parents and Children UK birth cohort, filtered, de-duplicated and cleaned it to generate a bibliographic research data set. This dataset could be used for accurate reporting and monitoring of the impact of the study as well as bibliometric research.

## Introduction

Birth cohort studies in the U.K. generate and distribute huge amounts of longitudinal data for medical, social and economic research. Data is generally applied for and given out to researchers once the relevant governance conditions have been met ^1^. It is often the case that these studies keep track of the publications that have arisen from the data they have given to researchers for project monitoring purposes and to report back to the funder(s). The size of these lists of publications is sometimes used as a crude metric of the the research outputs or impact for the study.

Most modern academic journals will assign a unique persistent identifier to new publications. This persistent identifier may be unique and resolvable by the journal, but may be meaningless outside of the journal’s ecosystem. The Digital Object Identifier (DOI) is the de facto persistent identifier which is used as an independent external reference to publications, posters, data, software etc. DOI resolving services exist to refer users (human and machine) to the journal web page for a given DOI, CrossRef holds over 100 million such records. These resolving services also host a wealth of metadata themselves. The DOI data model outlines the format of DOI data. In addition to CrossRef there exists other resolving and metadata services that are domain-specific. These may have more in depth metadata about their domain than the generality that the DOI data model can offer. In this work we also make use of the persistent identifiers that the National Center for Biotechnology Information (NCBI) PubMed generates (PubMed IDs, PMID), and the metadata their resolving service provides ^2^. This offers extra metadata over and above that available from CrossRef, but only on medical focused publications, i.e. a subset of all available publications in birth cohort studies.

In this paper we describe how we created a cleaned, de-duplicated list of peer-reviewed publications arising from the Avon Longitudinal Study of Parents and Children (ALSPAC). ALSPAC began in 1990 (see the cohort profiles for an overview ^3, 4^), and has publications within the biomedical research domain. ALSPAC reports to have over 1800 publications as of August 2018 ^5^. The study website contains details of all the data that is available through a fully searchable data dictionary and variable search tool.

## Methods

## Data cleaning

The ALSPAC master list of publications at the time this project started (2014), consisted of a large table in a Microsoft Word document. This table was imported into a spreadsheet containing a reference to the publication, a DOI and a PMID. Given the amount of time that has passed since the original master list was parsed we have merged this list with the list of publications on the ALSPAC website as at 12/9/18. One pertinent point is that there exists a small number of publications in the original Microsoft Word document that are not present on the website; we include these here for completeness.

Each publication was audited manually to ensure it was a peer reviewed publication i.e. that the journal had a defined peer-review process and/or that it appeared in Ulrichs Web Global Serials Directory with a "refereed" status. Non-peer-reviewed articles were removed from the publications list. Examples of non-peer-reviewed publications included theses, book chapters, published abstracts, opinion articles, comments on other articles, working papers and technical reports.

The DOI and PMID for each entry were also audited to validate the identifier and ensure they corresponded to the correct article. A common error was the truncation of a PMID, which due to the numerical nature of PMIDs was itself a valid PMID albeit referring to the wrong publication. If a DOI or PMID was missing from a publication, wherever possible this was sourced from the journal or PubMed directly. The DOI and PMID fields from the publications spreadsheet were used to import the publications lists into a bibliographic library in Zotero. Zotero uses NCBI PubMed to resolve PMIDs and CrossRef to resolve DOIs.

We then further cleaned the list of publications by deduplicating the list using Zotero’s native de-duplicate feature. Duplicates often arose in the bibliography when a publication was accepted in one year and then appeared online the next, or when it was listed with a DOI in one case and a PMID in another. Another common source of duplicates was having both the pre-print and the final published paper marked as separate items. In this case we disregarded the pre-print.

Given that publications are not necessarily reported to ALSPAC on acceptance to a journal, and some journals have a long turn around in publication time, we chose to have a cut-off of the end of 2015 for this data set. Given the misclassification of years of some publications, we added all publications up to the end of 2016 (as defined by the list on the ALSPAC website), but disregarded any that had a publication date after the end of 2015. This criteria left us with 1300 peer reviewed publications claimed by ALSPAC to the end of 2015. Table 1 shows a summary of the data.

**Table 1.** Data coverage. Percentages rounded down in each case.

Date range	1989–2015
Publication count	1300
DOIs (%)	97
PMIDs (%)	95
Publication title (%)	100
Year published (%)	100

## Data description

To make this list of publications available to others in as useful way as possible we exported it from our Zotero library in two different formats: BibTeX format to be able to import into any reference manager and comma separated variable (CSV) to allow import into analysis tools to do bibliometric analysis with. Both of these formats are described in Table 2 and Table 3, respectively. Zotero v5.0.56 was used to export the data.

**Table 2.** A data description of the BibTeX ALSPAC peer reviewed publications list to 2015.

Variable	Description
citation key	A unique identifier
title	Article title
author	Name(s) of author(s)
abstract	Article abstract
journal	Journal title
volume	Journal volume
number	Journal issue
pages	Article page numbers in the journal
year	Year published
month	Month published
keywords	Article keywords
issn	International Standard Serial Number
doi	Digital Object Identifier
pmid	PubMed identifier
pmcid	PubMed Central identifier

**Table 3.** A data description of the CSV file of ALSPAC peer reviewed publications list to 2015.

Variable	Description
Year	Year published
Author	Name(s) of author(s)
Title	Article title
Publication title	Journal title
ISSN	International Standard Serial Number
DOI	Direct Object Identifier
Abstract Note	Article abstract
Date	Date article published
Pages	Article page numbers in the journal
Issue	Journal issue
Volume	Journal volume
Extra	PubMed and/or PubMed Central ID;
Manual tags	Article keywords

## Data availability

The cleaned BibTeX and CSV data described here are available at Zenodo. DOI: https://doi.org/10.5281/zenodo.2276785 ^6^.

Data are available under the terms of the Creative Commons Attribution 4.0 International license (CC-BY 4.0).

All of the metadata presented here is publicly available in its raw form—the list of publications is available from the ALSPAC website and the individual publications’ metadata from their respective publishers. PubMed and CrossRef have additional terms and conditions ^1, 2^ on their aggregated metadata, but these are permissive and allow fair use.
