# Supplementary material for: Generation of a cleaned dataset listing Avon Longitudinal Study of Parents And Children peer-reviewed publications to 2015
Source: Wellcome Open Res. 2018 Dec 19;3:161. [Version 1] doi: 10.12688/wellcomeopenres.14986.1 (PMC6392145; doi:10.12688/wellcomeopenres.14986.1)
Supplement: Peer review history

# Peer review: Reviewer response for version 1

**DOI:** 10.21956/wellcomeopenres.16347.r34479

**Published:** 2019-02-22

**Authors:** Dylan Kneale

**Affiliations:** 1 Evidence for Policy and Practice Information and Coordinating Centre, UCL Institute of Education, University College London (UCL), London, UK

Thank you for the opportunity of reviewing this data note. I think that this represents really exciting work and a huge effort in documenting studies using the data and cleaning these.

 There are some aspects that I think could be better described to help support other similar exercises in the future:

1. I think the rationale around collating this information could be strengthened a little. In particular, I think the rationale should better make the case that understanding the scientific impact of these cohort studies is key in supporting the continuation of this study and funding future studies.
2. The source data are drawn from records held by the ALSPAC team, which has been keeping track of publications. These formed a ‘master list’ of publications which was then extensively cleaned and refined to form the dataset. However, the processes used to keep track of publications need to be better described – how are studies identified and to what extent do the researchers feel that these records represent the full range of peer-reviewed studies published using ALSPAC data?
3. The authors described that these are publications arising from ALSPAC data. Were any criteria imposed on what this ‘usage’ should look like? For example would a commentary that makes reference to the ALSPAC data (possibly alongside other studies) be included as a publication; would secondary analyses of studies using ALSPAC data be included (e.g. using an effect size from a study using ALSPAC data as part of a meta-analysis)? While the PMID and DOIs were cleaned, were the studies checked for their usage of these data? This seems important to clarify. While this data note describes a dataset of ALSPAC publications, I’m not clear if this is exclusively a dataset of primary studies using ALSPAC data in novel analyses, or if it also includes other publication types. If the dataset does include other publication types, does this have implications for the way in which the dataset should be used by future researchers?
4. As a minor suggestion, it may be interesting to have an addition to Table 1 that includes a breakdown of publications by year.

 The suggestions made above are mainly for clarification to help understand the parameters of the data set. I would like to again emphasise that this data note does represent a huge task undertaken and has resulted in a very worthwhile output.

I have read this submission. I believe that I have an appropriate level of expertise to confirm that it is of an acceptable scientific standard.

# Peer review: Reviewer response for version 1

**DOI:** 10.21956/wellcomeopenres.16347.r34616

**Published:** 2019-01-29

**Authors:** Carly Strasser

**Affiliations:** 1 Fred Hutchinson Cancer Research Center (Fred Hutch), Seattle, WA, USA

The manuscript reports on creating a complete bibliography of publications associated with the ALSPAC longitudinal study. Collecting such data is not trivial given the duplication via preprints, PMIDs, and variable metadata associated with articles. This work is important for understanding the impacts of the study, as well as potential future meta-analyses.

I have read this submission. I believe that I have an appropriate level of expertise to confirm that it is of an acceptable scientific standard.
